# In silico design of targeted SRM-based experiments

**DOI:** 10.1186/1471-2105-13-S16-S8

**Published:** 2012-11-05

**Authors:** Sven Nahnsen, Oliver Kohlbacher

**Affiliations:** 1 Center for Bioinformatics, Quantitative Biology Center, and Department of Computer Science, University of Tübingen, Germany

## Abstract

Selected reaction monitoring (SRM)-based proteomics approaches enable highly sensitive and reproducible assays for profiling of thousands of peptides in one experiment. The development of such assays involves the determination of retention time, detectability and fragmentation properties of peptides, followed by an optimal selection of transitions. If those properties have to be identified experimentally, the assay development becomes a time-consuming task. We introduce a computational framework for the optimal selection of transitions for a given set of proteins based on their sequence information alone or in conjunction with already existing transition databases. The presented method enables the rapid and fully automated initial development of assays for targeted proteomics. We introduce the relevant methods, report and discuss a step-wise and generic protocol and we also show that we can reach an *ad hoc *coverage of 80 % of the targeted proteins. The presented algorithmic procedure is implemented in the open-source software package OpenMS/TOPP.

## Introduction

Mass spectrometry (MS) has become the most important method for protein identification and quantitation. In shotgun proteomics proteins are usually digested into smaller peptides. The complex mixture of peptides is then analyzed with high-performance liquid chromatography (HPLC) coupled to a mass spectrometer (LC-MS). The fragmentation of peptide ions allows the determination of the sequence by recording production masses. This method is called tandem MS [1] and is an established method in many laboratories. The selection of peptide ions for fragmentation in tandem MS is most commonly done in a data-dependent acquisition (DDA) where the *n *most abundant precursor ions are selected for fragmentation in each survey scan. Coupled with efficient separation methods, DDA allows in-depth proteome analysis. Due to the stochastic nature of ion sampling, DDA is accompanied with bad reproducibility and not uncommonly low-abundant proteins remain unseen.

**Targeted proteomics **based on selected reaction monitoring (SRM), in contrast, is a popular technology that avoids some of the drawbacks of DDA-based shotgun proteomics. SRM-based analysis of protein expression has been shown to be highly sensitive [2]. Sensitivity, dynamic range and reproducibility of SRM assays are increased compared to a shotgun assay, therefore, SRM is a promising tool for clinical applications, especially for biomarker validation in blood plasma [3]. In contrast to DDA methods, SRM-based proteomics targets only selected proteins/peptides and thus relies on knowledge of the selected precursor and their productions. Peptides are monitored using *transitions*. A transition is defined as the pair of precursor mass/charge ratio and a production mass/charge ratio. Technically, these transitions are measured on a triple-quadrupole mass spectrometer, which enable to selectively choose precursor ions in the first quadrupole, trigger their fragmentation in the second quadrupole and monitor specific fragment ions in their quadrupole mass analyzer. The ability to quantify proteins is comparable to western blotting or ELISA assay, but much easier to parallelize, automate, and replicate. In classical SRM experiments those transitions have been constructed based on knowledge from previous experiments [4]. Although SRM-based methods cannot be used for discovery approaches, such experiments play an increasingly important role for biomarker validation and quantitative studies in systems biology, where researchers are interested in quantitative information for specific pathways only. SRM has also been successfully applied to large-scale genome-wide experiments [5,6]. In [6] the authors show that SRM assays are capable to cover the full dynamic range of protein expression of small sized eukaryotic organisms, such as *S. cerevisiae*.

**The selection of SRM transitions **remains a difficult task. Transition information based on experimental data on a variety of different biological and technical systems, is accumulated in databases. A commonly used repository for SRM transitions is the MRM atlas webpage (http://www.mrmatlas.org/) and several tools emerged that make use of known information to design SRM assays. Examples for SRM design platforms include Skyline [7,8], as well as MRMaid [9,10]. Such tools have become indispensable in the design for targeted proteomics experiments. While these tools allow for an efficient construction of transition information from public data and also for the prediction of retention times for the individual peptides, the optimal schedule of transitions with the objective to maximize protein identifications needs the formulation of an optimization problem in addition. Furthermore, the heterogeneity of data sets, instrumental conditions and the focus on human samples and some model organisms, limit the applicability of the data repositories as a single source for transition information. Targeted SRM experiments greatly benefit from both, the optimal selection and the optimal scheduling of transitions. It is thus desirable to use a semi-automated integration of SRM design tools, such as MRMaid and Skyline, with our solution to the optimization problem as described below. Generally one should select only peptides that can be uniquely mapped to a protein and that are detectable in a mass spectrometer. Therefore the notion *proteotypic peptide *is frequently used in SRM-based proteomics [11,12]. A *proteotypic peptide *is unique for a protein with respect to a given proteome and detectable through the mass spectrometer. Proteotypicity is thus an extension of the commonly used peptide detectability property to the unique mapping of the peptide to a single protein. Peptide detectability has been shown to be a crucial parameter for protein quantification and identification [13,14]. In principle, these peptides can be systematically determined for all proteins of an organism; however, this approach is rather expensive. There have been numerous approaches that suggest computational methods for the prediction of peptide proteotypicity/detectability [14] and in 2007, the notion of proteotypicity was introduced [11]. Given the research effort put into the computational prediction of peptide detectability, it is possible to construct SRM assays *de novo*, that is, from the protein sequences alone. If transition information is present for the given organism and instrument, this information can be incorporated into the assay, additionally to the *de novo *constructed transitions. In SRM, a mass filter selects the precursor m/z value and after CID a specific production m/z value is monitored. The number of transitions that a mass spectrometer can monitor in parallel is limited, currently, to a few dozen transitions at best. Peptides typically elute over short time spans only, hence the transition needs to be monitored only within a small retention time window. Restricting the monitoring of each transition to a limited time window can thus increase the overall assay capacity tremendously, but it requires knowledge of the peptide retention time. Given a large number of transitions of different peptides/proteins, the transitions need to be arranged in an experiment, such that the measurement time is used efficiently. Even in low-complexity samples there are many more transitions that could be scheduled compared to the overall measurement time.

**Figure 1:**
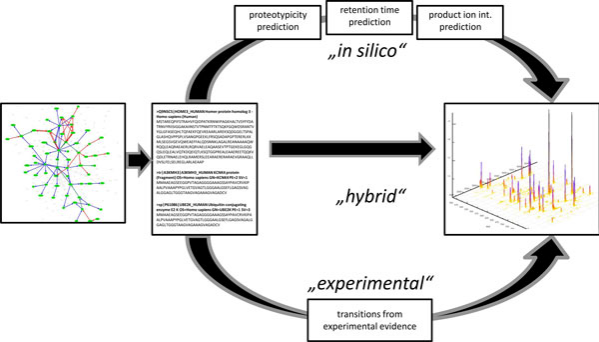
**Overview**. The basis of an SRM experiment are the targeted proteins (here proteins involved in a pathway). For those protein sequences the SRM transitions can be determined experimentally, they can be found using prediction methods or a hybrid method is used, where transitions are partly predicted and retrieved from repositories. Regardless of their calculation, the transitions are then scheduled and the mass spectrometric data is acquired.

**An optimization problem **can be formulated to select the best set of transitions: how to choose between thousands of transitions such that the number of proteins that are observed is maximized and the error in protein quantification minimized? We introduce a novel method for the optimal *de novo *design of targeted SRM experiments based on the protein sequences alone or in conjunction with existing transition information. Apart from a simple calibration run (e.g., a protein mix) to determine the properties of the chromatographic system, no further experimental data is required. Our approach is based on machine learning methods and combinatorial optimization. Machine learning methods predict peptide proteotypicity [11,13,15], peptide retention times [16,17], and suitable productions for SRM transitions (see upper path in Figure 1). These peptide properties may also be determined experimentally (see lower path in Figure 1). Besides the pure computational design of the targeted proteomics experiment, we also illustrate how existing, experimentally determined transition can be incorporated into the formulation of the optimization problem. From the total set of suitable transitions, we then formulate the *SRM scheduling problem*, which optimizes the measurement schedule with respect to protein and peptide coverage while ensuring that each peptide is covered by a minimal number of SRM transitions. At the same time, an optimal design also makes the best use of instrument measurement time by scheduling as many transitions as possible. We describe training and evaluation of the prediction methods used in this work. The scheduling problem is formally described as an integer linear program (ILP) [18]. Despite the complexity of the problem, we found that most real-world instances of the problem can nevertheless be solved in acceptable time. We show the performance and applicability of these methods on a simple example, where we generate a scheduled SRM assay for a protein mixture and we outline the integration of experimentally determined transitions. For the pure computational approach (without the integration of experimental data), we can show that as expected from the performance of single prediction methods, about half of the transitions work without experimental validation. The resulting SRM schedule is thus an excellent starting point for subsequent experimental optimization.

## Materials and methods

### OpenMS

OpenMS is a comprehensive C++ framework for computational mass spectrometry. A wide range of MS-related data structures and algorithms allow rapid prototyping of data processing pipelines for mass spectrometry-based proteomics. OpenMS is freely available at www.openms.de. All experiments outlined here can be performed using the OpenMS library.

### Data generation

All data used in the example processing pipeline have been acquired with the same instrumental setup. A mouse proteome dataset was used as training data and the UPS1 protein mixture, containing 48 different proteins (Sigma Aldrich, Catalog Number U6133) was used as test data. All protein mixtures were digested with trypsin (Promega) and the resulting peptide mixtures were analyzed using a nanoflow LC (Proxeon Biosystems) with nano-HPLC column (75 mm by 15 cm) packed in-house with 3-mm C18 beads (Dr. Maisch). The LC was online coupled to a 4000 QTrap (ABSciex). The mass to charge range for precursor selection for Q1 was set to 400-1000 Th (Thomson) and for production selection for Q3 to 400-1,200 Th.

#### Training data

Peptide identifications for the mouse dataset were performed using consensus identifications [19] on the basis of the Mascot [20], X!Tandem [21] and OMSSA [22] search engines. The SwissProt [23] mouse database (version 57.1) was used for all database search programs. All searches were performed using a combined target/decoy database. The decoy sequences were generated by reversing all protein sequences. False discovery rates were estimated using q-values [24] and a q-value cut-off of 0.01 was used to extract correct identifications. Carbamidomethylation of cysteines was set as a fixed modification and no variable modifications were allowed. The precursor mass tolerance was set to 0.8 Da and the production tolerance was set to 0.5 Da. For the different prediction models the datasets were created as follows:

If the same sequence and charge was identified several times, the spectrum with the highest total ion current (TIC) was kept. The best 1,000 peptides (ranked according to their q-values) were used for training of the retention time and the proteotypicity. No missed cleavages were allowed for the training. The proteotypicity model needs additional negative examples; peptides that have theoretical m/z values within the instrument detection range, but are not observed in the training dataset. One thousand undetected peptides from proteins with high sequence coverage (many identified peptides) were used for this purpose.

#### Experimental test data

The transitions were calculated and optimally scheduled for the 48 proteins included in the UPS1 mix. The experimental processing was done as for the training data.

#### Pre-existing, experimentally validated transition information

Besides the pure computational determination of the optimal transition schedule, our approach also allows additional incorporation of information from existing repositories. In this article, we illustrate the information retrieval for the SRMAtlas (http://www.srmatlas.org) repository. The data retrieval is from public repositories is exemplified with the *α*-lactalbumin protein (UniProtKB/Swiss-Prot: P00709 (LALBA_HUMAN)).

### Algorithmic procedures

The aim of targeted proteomics experiments is the identification and quantification of a given set of proteins. The size of this set can range from single proteins, such as biomarkers, over moderate sized sets, such as all components of a cellular pathway, up to the entire proteomes. Depending on this target protein set the formulation of the problem needs information on the proteotypicity and the retention time of all theoretical peptides that can result from the protein sequences, as well as on the production intensity for a given peptide sequence.

These unknowns can either be filled by experimental evidence, by *de novo *calculation of prediction models (see Figure 1) or by a combination of both. In the following we will summarize the methods underlying the single prediction methods and finally we will show the formulation of an integer linear program that allows combining different methods to optimally schedule transitions in an SRM assay. The final combination needs to cope with the uncertainty from the prediction methods, while optimizing the coverage of the proteins that are analyzed within one experiment.

#### Integer linear program (ILP)

An ILP is a technique for the optimization of a linear objective function. For the SRM experimental design, this optimization problem is to find the maximal number of transitions that can be placed into one experiment, while preserving several constraints, such as the number of simultaneous transitions.

#### Retention time prediction

For the prediction of retention times for peptide sequences a model is calculated based on support vector regression (SVR [25]). The relevant sequence information is integrated in this model using a specialized string paired oligo border kernel (POBK). A detailed algorithmic description of this method can be found in [16]. The training of the retention time model needs data that were acquired on the same instrument as the experiment. The method shown here requires only about 40 peptides with accurately annotated retention times. The support vector regression method aims to find a function *f *: *X *→ *Y*, *Y *⊆ ℝ from *n *labeled training samples (*x_i_*, *y_i_*) ∈ {(*x_i_*, *y_i_*) *|x_i _*∈ *X*, *y_i _*∈ *Y*, *i *= 1, .., *n*} in order to allow predictions *y *∈ *Y *to unknown data samples *x *∈ *X *from the same data source.

#### Proteotypicitiy prediction

Proteotypic peptides are unique and detectable peptides. While uniqueness within a given database is trivial to determine, prediction of detectability is less trivial. It is common knowledge that not all peptides of a digested protein are detectable in an LC-MS experiment [15]. Many different physicochemical properties of peptides have impact on ionization efficiency during electrospray ionization (ESI).We integrated an additional machine learning-based model for peptide proteotypicity prediction. In this context, proteotypicity refers to the peptide detectability in the mass spectrometer. For this prediction a support vector-based approach in combination with a tailor-made kernel function was chosen that is similar to the model used for peptide retention time prediction. The training dataset includes positive (detectable peptides) and negative (non-detectable peptides) examples. Good performance is observed if at least 1,000 positive as well as 1,000 negative examples are chosen for training. These training examples can be easily extracted from the identification results of existing shotgun proteomics runs. If the training data has been identified using the target-decoy approach [26], peptide identification with q-values ≤ 0.01 are chosen as positive examples. Negative examples are chosen by selecting non-observed tryptic peptides with appropriate theoretical m/z values from proteins that were identified with sufficient sequence coverage. For all experiments we set the protein sequence coverage to be at least 15 %.

#### Fragment intensity prediction

An accurate prediction of fragment spectrum intensities allows selecting the most intenseions and thus the most sensitive SRM transitions. For the prediction of fragment ion intensities OpenMS implements a hidden Markov model based on the mobile proton hypothesis and the main peptide fragmentation pathways [27]. This model generates a theoretical fragment spectrum including fragment ion intensities for any given peptide sequence and charge. For the selection of the optimal transitions we employed several criteria, such as a limited production mass range; fragment masses are not used if they are likely to interfere with others and the predicted retention time has to be in an appropriate range.

#### Retrieval of pre-existing transition information

**Figure 2:**
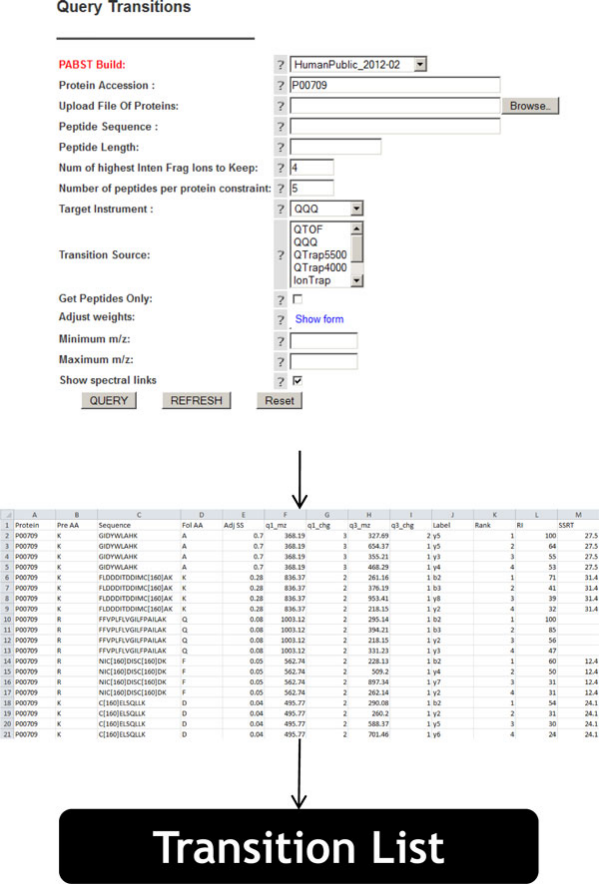
**Transition retrieval from www.srmatlas.org**. The user interface of SRM atlas allows to query for transitions of proteins of interest. Here transitions were retrieved for the *α*-lactalbumin protein (P00709).

The hybrid approach, as visualized in Figure 1, needs information that is stored in public repositories. Figure 2 illustrates the information that can be manually retrieved from the the PeptideAtlas repository. Once the information is downloaded, it can be incorporated into or replace the list of possible transitions that has been created via machine learning tools. The downstream formulation of the optimization problem remains the same, independent of whether experimentally confirmed or *in silico *predicted transition lists (or even a mix thereof) are used.

**Figure 3:**
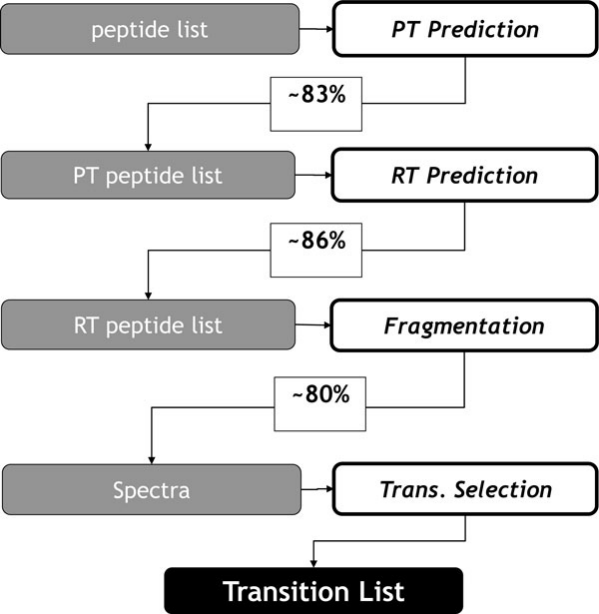
**Computational prediction pipeline**. The left panel shows the input data for the prediction tools (on the right) as implemented in OpenMS. The performance of the method is indicated after each tool. The selection of transitions (Trans. Selection) is performed by finding solutions to the ILP.

#### Optimal experimental design

The problem formulation assumes that we are given a fixed set of protein sequences. Furthermore, we need prediction models for (i) proteotypicity, (ii) retention time, and (iii) production intensities for a given peptide sequence. If prediction models were not available, those properties need to be determined experimentally. Although prediction models have a limited accuracy, we have previously shown that the prediction of these models is by far accurate enough to enable *ab initio *construction of SRM assays [27].

The model optimization problem can then be formulated as follows:

We are given *k *protein sequences *S *= {*s*_1_, *...s_k_*}. For these sequences we assume that each proteins contains at least one tryptic peptide. The union of all peptides is given by *P *= {*p*_1_, *.*.., *p_m_*} and for each peptide we can predict the retention time *RT *(*p_i_*), the proteotypicity *PT *(*p_i_*) and a list of production intensities *FI*(*p_i_*). In order to maximize the number of transitions observed in a single LC-MS run, the transitions have to be scheduled according to the peptide's elution time. The set of all possible transitions is denoted as *T *= {*t*_1_, ..., *t_l_*}, where each transition *t *consists of a peptide ion mass/charge value *p*(*t*) and a production mass/charge values *m*(*t*). The proteotypicity of the precursor ion and the corresponding fragment ion intensity for the transition *t*, will be combined in the joint detectability *d_t_*.

**Figure 4:**
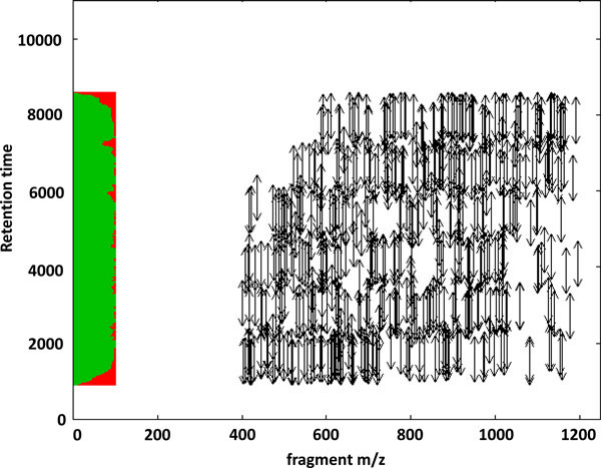
**Theoretical 2D transition map**. The arrows indicate the tolerance that is allowed for the predicted retentions times. The color scheme next to the y-axis corresponds to the occupancy of the acquisition time. We allowed only masses between 400 and 1200 Th.

Based on a peptide's retention time we can reserve time slots of length 2*δ *(where *δ *denotes the retention time tolerance allowed) for each transition. The resulting scheduling problem is illustrated in Figure 4. The *SRM scheduling problem *can then be formulated as an integer linear program by introducing:

We introduce binary decision variables *x_t _*with

$$x_{t}=\left\{\begin{array}{c} 1,\;\;\;\text{if}\;\text{transition}\;t\;\text{is}\;\text{in}\;\text{the}\;\text{schedule}\\ \text{0,}\;\;\;\text{otherwise} \end{array}\right)$$

Furthermore we introduce variables *y_p _*for each peptide *p *∈ *P *with

$$y_{p}=\left\{\begin{array}{c} 1,\;\;\;\text{if}\;p\;\text{is}\;\text{not}\;\text{covered}\;\text{by}\;\tau \;\text{transition}\\ \text{0,}\;\;\;\text{otherwise} \end{array}\right)$$

and for each protein sequence *s *∈ *S *we define *ρ *binary variables $z_{s}^{j}$ with

$$z_{s}^{j}=\left\{\begin{array}{c} 1,\;\;\;\text{if}\;s\;\text{is}\;\text{not}\;\text{presented}\;\text{by}\;j\;\text{peptides}\\ \text{0,}\;\;\;\text{if}\;\text{covered}\;\text{by}\;\text{at}\;\text{least}\;j\;\text{peptides} \end{array}\right)$$

Additionally, two constants, *ω^p ^*and *ω^s ^*are introduced. The objective function is penalized by *ω^p^*, if a peptide *p *is not covered by at least *τ *transitions. In a similar *ω^s ^*is used to penalize, if a protein *s *is not covered by at least *j *peptides. A reasonable choice is one and ten for *ω^p ^*and *ω^s^*, respectively. With the binary decision variables and the two constants, we can formulate the scheduling problem as follows:

(1) $$\text{max}\text{imize}\underset{t\in T}{\sum }x_{t}d_{t}-\omega^{s}\underset{p\in P}{\sum }y_{p}-\omega^{s}\underset{s\in S}{\sum }\underset{0\leq j\leq \rho }{\sum }\overset{j}{\underset{s}{z}}{\left(\rho -j\right)}^{2}$$

subject to

(2) $$\tau y_{p}+\underset{i\in Tp}{\sum }x_{i}\geq \tau ,\forall \;p\;\in \;P$$

(3) $$\begin{array}{c} \left(j+1\right)z_{s}^{j}+\underset{p\in P_{s}}{\sum }tcov\left(p\right)\geq j+1,\forall \;s\in S,\forall \;0\leq j<\rho \\ \text{with}\;tcov\left(p\right)=\left\{\begin{array}{c} 1,\;\;\;\Sigma_{i}\;x\geq \tau \\ 0,\;\;\;\text{otherwise} \end{array}\right) \end{array}$$

(4) $$\underset{j\in TS_{i}}{\sum }x_{j}\;\leq C,\forall \;1\leq i\leq \;N$$

The first constraint (2) is introduced to ensure that each peptide is covered by at least *τ *transitions. Note that this constraint is fulfilled if there are *τ *transitions for peptide *p *or *y_p _*equals 1. Similarly, the next constraint (3) ensures that each protein is covered by at least *ρ *peptides. The final constraint (4) limits the number of transitions that are scheduled in parallel to at most *C*. The ILP, defined by eqs. (1)-(4), was implemented in C++ based on the GNU Linear Programming Kit (GLPK) and is available as part of OpenMS.

## Results and discussion

### SRM assay design

The SRM assay can be set up in step-by-step fashion: Initially, the set of targeted proteins needs to be chosen. This set can include only one protein (e.g., for the validation of a single biomarker) or it could be a larger set of proteins involved in a common pathway. All subsequent predictions will be based on these protein sequences. In our example pipeline this set contains all proteins from the Sigma UPS1 mix. In order to have enough data points for the machine learning, a training dataset needs to be acquired. It is important to consider significant changes in retention time if the HPLC column has been changed between runs. If this is the case, it might be necessary to acquire new training data. The training data should ideally include several hundreds to a few thousands non-redundant peptides. In our example this training dataset has been acquired on whole-proteome measurements from mouse kidney tissues. Following the acquisition of the training data, these can be used to train the models. The OpenMS library provides an easy interface for the training of models for proteotypicity, retention time, and fragment ion intensity prediction, but other tools can also be incorporated via the wrapping functionality of OpenMS. As outlined in the methods section we used the significantly identified peptides from the mouse data for the training of all models. With these models, a scheduled SRM experiment can be designed. If the hybrid approach is taken and parts of the transitions have been downloaded from public repositories, this information is simply incorporated in the transition list before the optimization is run on the final list. At any point during the generation of the initial list data can be exchanged in a semi-automated fashion with other design tools, such as Skyline or MRMaid via the open standard transition exchange format *TraML *[28]. Interestingly, many of the precursor/production pairs that are suggested by the machine learning algorithms are also suggested by the repositories. Optimal solutions to the scheduling problem allow to determine the information that is necessary to write the transition lists. At this point, there is no difference between transitions that originate from pure computational prediction and transitions that were extracted from repositories.

### Accuracy of in silico predictors

**Figure 5:**
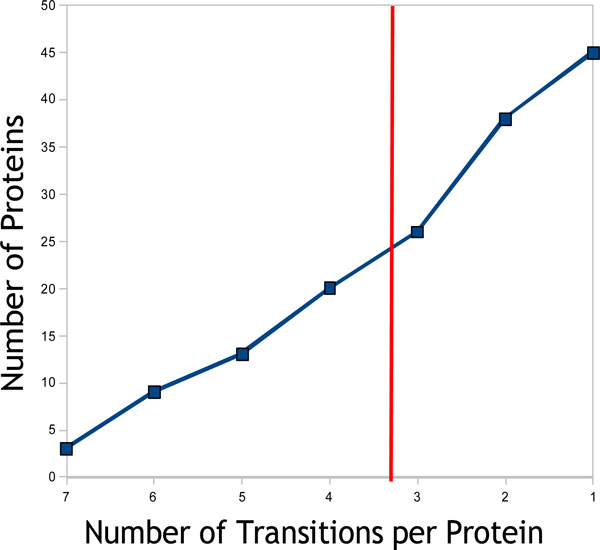
**Number of transitions per protein**. In our example experiment we analyzed a protein mixture of 48 proteins. Here we show the number of transitions that we obtained for the 45 proteins that were detected in our experiment.

The overall algorithmic framework of the *in silico *prediction method described here is shown in Figure 3. As each prediction method has only limited accuracy, we calculated the prediction performance for each prediction method independently in order to estimate the overall accuracy for our final SRM transition list. The complete SRM transition calculation can be approximated by an independent combination of probabilities of correct predictions as determined in a cross-validation.

$$P\left(\text{measured transition}\right)=0.\text{83}\;\times \;0.\text{86}\;\times \;0.\text{8}0=0.\text{57}$$

is thus an estimate of how probable a transition will be measured by a given mass spectrometer. Our experiments support this number: about half of the predicted transitions can be observed in the experiment. With a sufficiently large number of peptides/transitions per protein this still implies that the vast majority of the proteins can be quantified. Using these lists, we detect 45 out of 48 proteins by at least one transition and have an average of 3.2 transitions per protein as shown in Figure 5. The raw data can be requested from the others and the software source code is freely available at www.openms.de.

### Optimal usage of instrument time

Solutions to the scheduling problem can be found, using the OpenMS implementation that is part of the current development version. Figure 4 shows a typical schedule for transitions. Solutions to the ILP produce a transition list that ensures a minimum number of transitions per protein, a maximized number of transitions to increase coverage as well as accuracy, and an optimal use of instrument acquisition time. In our example experiment we can fill up to 92% of all possible time slots during the HPLC gradient.

## Conclusions

Targeted proteomics aims at the accurate and reproducible detection of a predefined set of proteins. Selection reaction monitoring (SRM) is the method of choice in most targeted proteomics experiments. We present an algorithmic procedure that enables the construction of SRM transitions given the protein sequences of the targeted proteins only. Despite limited accuracy of prediction methods, the approach yields good initial transition lists that allow the quantification of the vast majority of the targeted proteins even without subsequent experimental optimization. It does not rely on data repositories or the experimental determination of SRM transitions and can automatically adapt to any experimental setup through the use of machine learning methods.

## Competing interests

The authors declare that they have no competing interests.

## Authors' contributions

SN developed the methods and wrote the manuscipt. OK designed the study and wrote the manuscript.
